# Population structure and uropathogenic potential of extended-spectrum cephalosporin-resistant *Escherichia coli* from retail chicken meat

**DOI:** 10.1186/s12866-021-02160-y

**Published:** 2021-03-29

**Authors:** May Linn Buberg, Solveig Sølverød Mo, Camilla Sekse, Marianne Sunde, Yngvild Wasteson, Ingun Lund Witsø

**Affiliations:** 1 Department of Paraclinical Sciences, Faculty of Veterinary Medicine, Norwegian University of Life Sciences, Oslo, Norway; 2 Section for Food safety and Animal Health Research, Department of Animal Health and Food Safety, Norwegian Veterinary Institute, Oslo, Norway

**Keywords:** Antimicrobial resistance, AMR, Poultry, Foodborne, Norway, Phenotype, *E. coli*, Virulence, Urinary tract infection, UTI

## Abstract

**Background:**

Food-producing animals and their products are considered a source for human acquisition of antimicrobial resistant (AMR) bacteria, and poultry are suggested to be a reservoir for *Escherichia coli* resistant to extended-spectrum cephalosporins (ESC), a group of antimicrobials used to treat community-onset urinary tract infections in humans. However, the zoonotic potential of ESC-resistant *E. coli* from poultry and their role as extraintestinal pathogens, including uropathogens, have been debated. The aim of this study was to characterize ESC-resistant *E. coli* isolated from domestically produced retail chicken meat regarding their population genetic structure, the presence of virulence-associated geno- and phenotypes as well as their carriage of antimicrobial resistance genes, in order to evaluate their uropathogenic potential.

**Results:**

A collection of 141 ESC-resistant *E. coli* isolates from retail chicken in the Norwegian monitoring program for antimicrobial resistance in bacteria from food, feed and animals (NORM-VET) in 2012, 2014 and 2016 (*n* = 141) were whole genome sequenced and analyzed. The 141 isolates, all containing the beta-lactamase encoding gene *bla*_CMY-2_, were genetically diverse, grouping into 19 different sequence types (STs), and temporal variations in the distribution of STs were observed. Generally, a limited number of virulence-associated genes were identified in the isolates. Eighteen isolates were selected for further analysis of uropathogen-associated virulence traits including expression of type 1 fimbriae, motility, ability to form biofilm, serum resistance, adhesion- and invasion of eukaryotic cells and colicin production. These isolates demonstrated a high diversity in virulence-associated phenotypes suggesting that the uropathogenicity of ESC-resistant *E. coli* from chicken meat is correspondingly highly variable. For some isolates, there was a discrepancy between the presence of virulence-associated genes and corresponding expected phenotype, suggesting that mutations or regulatory mechanisms could influence their pathogenic potential.

**Conclusion:**

Our results indicate that the ESC-resistant *E. coli* from chicken meat have a low uropathogenic potential to humans, which is important knowledge for improvement of future risk assessments of AMR in the food chains.

**Supplementary Information:**

The online version contains supplementary material available at 10.1186/s12866-021-02160-y.

## Background

*Escherichia coli* is a highly diverse species that includes commensals, pathogens, and opportunistic pathogens. *E. coli* that cause infections outside the intestinal tract are commonly referred to as extraintestinal pathogenic *E. coli* (ExPEC). ExPEC is usually phenotypically indistinguishable from gut-colonizing commensal *E. coli.* Based on their virulence traits, they are often divided into subgroups such as uropathogenic *E. coli* (UPEC), isolates causing septicemia, neonatal meningitis-causing *E. coli* (NMEC), and avian pathogenic *E. coli* (APEC) [1, 2]. As ExPEC are mainly considered opportunistic pathogens, it has been challenging to define a set of virulence factors for this group of bacteria. Terms like “fitness factors”, “colonizing factors”, and “virulence-associated traits” have been suggested as being more accurate for describing specific traits that distinguish ExPEC from other *E. coli* [2–4]. Johnson et al. proposed a list of ExPEC virulence-associated traits which included various adhesins, toxins, nutritional characteristics, protectins, and miscellaneous traits [3]. The common denominator for all these traits is provision of competitive advantages and survival outside the intestinal tract, with potential to cause disease in various other tissues [2–4]. Several reservoirs for ExPEC have been described, for example the intestinal tract of humans, companion animals, and food-producing animals [5]. Different typing methods have been applied for epidemiological purposes and understanding of the transmission of ExPEC between different reservoirs and hosts, allowing for differentiation of *E. coli* into group levels. Multilocus sequence typing (MLST) groups *E. coli* into various sequence types (STs), and some STs are known to possess a higher pathogenic potential than others [6].

Urinary tract infection (UTI) is one of the most common bacterial infections encountered in the human population worldwide, and comes with great societal costs [7]. A UTI starts with bacteria, such as UPEC, colonizing the distal parts of the urethra, thereby ascending into the bladder, adhering to the surface of the bladder, followed by biofilm formation, and then invasion and replication within the hosts cells [8–10]. Both structural and secretory features are involved in UPECs ability to cause UTI [8]. Structural virulence-associated traits including adhesins, fimbriae, flagella, and other surface components are involved in colonization of the mucosal surfaces in the urinary tract, while secreted components, such as toxins and enzymes, are responsible for cell-damage [11]. UTIs can vary from a mild bacteriuria to severe urosepsis, and antimicrobials are often needed for curing the infection [12].

Antimicrobial resistance (AMR) is one of the largest threats against global public health in our time [13, 14]. Use of antimicrobials is regarded as the most important driver for development and dissemination of AMR, although the exact and relative amounts distributed to and between human and veterinary medicine vary considerably from country to country [15–17]. On a global basis, the use and overuse of antimicrobials in food-producing animals is extensive, and the co-occurrence of AMR, including extended-spectrum cephalosporin (ESC)-resistant *E. coli,* in the food chains is considerable [15, 17, 18]. The Norwegian monitoring program for antimicrobial resistance in bacteria from food, feed, and animals (NORM/NORM-VET) have for several years documented that Norway is among the European countries with the lowest levels of antimicrobial use and corresponding low levels of AMR [13, 14, 19, 20]. NORM-VET is governed by the legislation ensuring harmonized AMR monitoring within the EU, and poultry are sampled every other year [21]. Results document that the Norwegian broiler production has a low level of antimicrobial use with only one to seven flocks treated yearly between 2013 and 2017 [22–27]. ESC-resistant *E. coli* have nevertheless been detected in healthy broilers and retail chicken meat using selective methods since their first observed appearance in 2006, and with significant reduction since 2012 [28, 29].

Food-producing animals and their products are considered a possible source for human acquisition of AMR *E. coli* [30–32]. ESC-resistant *E. coli* isolates are of particular interest, as extended-spectrum cephalosporins are listed as critically important antimicrobials by the World Health Organization [33]. Genetic comparisons of *E. coli* isolates from poultry and clinical UPEC isolates have been observed to have a high degree of similarity [34], and *E. coli* isolates from meat have also been shown to cause UTI in murine models [35]. In a review from 2015, Lazarus et al. considered poultry to be the most likely source of human acquisition of ESC-resistant ExPEC from food-producing animals [36], and consumption of chicken meat could thus be a possible route of ExPEC transmission, including UPECs [35–38]. In a report from 2015 the Norwegian Scientific Committee for Food and Environment (VKM) also concluded that poultry and poultry products are regarded as the most important reservoirs of ESBL/AmpC-producing *Enterobacteriaceae*, quinolone resistant *E.coli* (QREC), and their corresponding resistance determinants [39]. However, lack of data has made it difficult to reach any firm conclusions regarding the probability of AMR transmission from food to humans.

The aim of this study was to characterize ESC-resistant *E. coli* isolated from domestically produced retail chicken meat regarding their population genetic structure, the presence of virulence-associated geno- and phenotypes as well as their carriage of AMR genes, in order to evaluate their uropathogenic potential. To ensure a highly relevant collection of bacterial isolates, all ESC-resistant *E. coli* isolates collected from retail chicken meat through NORM-VET in 2012, 2014, and 2016 were included in the initial screening and description. Representative isolates were selected for further phenotypic virulence characterization.

## Results

### Characterization of population structure

**Fig. 1:**
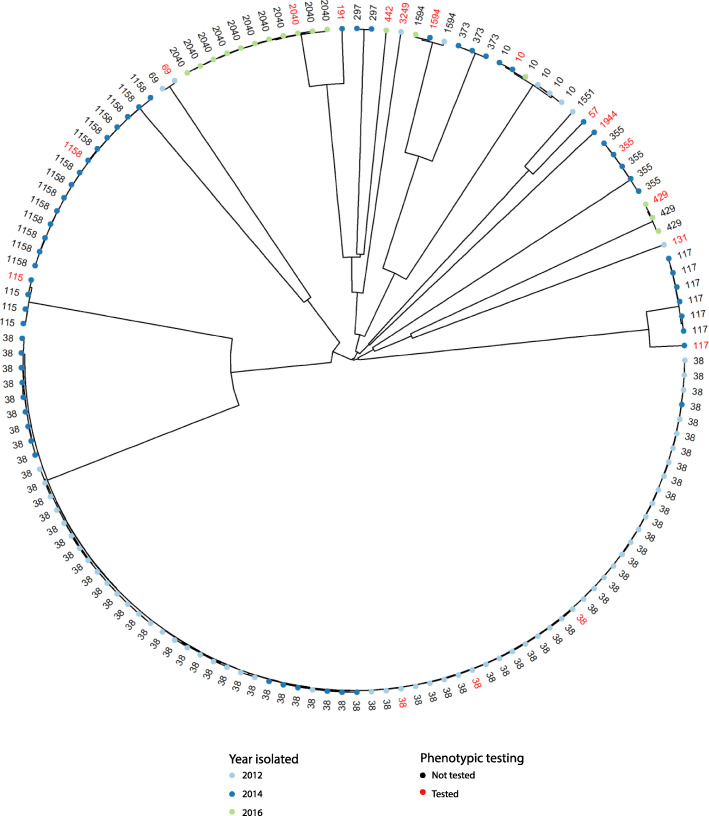
Clustering of 141 ESC-resistant *Escherichia coli* isolated from Norwegian retail chicken meat. The clustering is based on core genome multilocus sequence typing. Year isolated is indicated as light blue (2012), blue (2014), or light green (2016) dots. The 18 isolates marked in red were included in the in vitro virulence assays

**Fig. 2:**
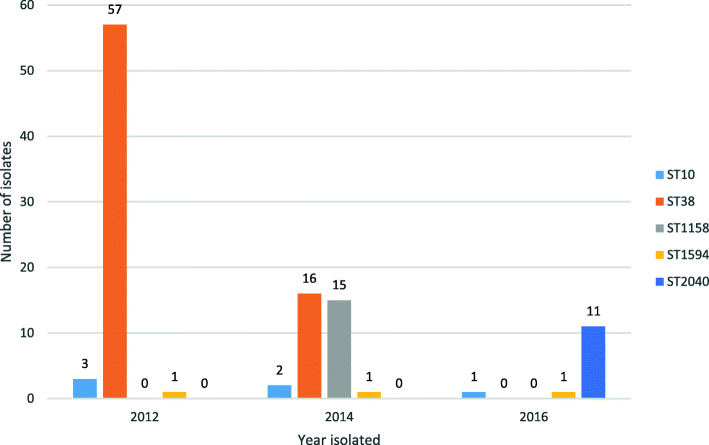
Fluctuation in selected sequence types during 2012, 2014, and 2016. The figure illustrates the distribution of *Escherichia coli* isolates belonging to the three most common sequence types, namely ST38, ST1158, and ST2040 in 2012, 2014, and 2016. Furthermore, the number of isolates grouping into ST10 and ST1594 is illustrated, as these were the only sequence types present in all three years

All the 141 ESC-resistant isolates were whole genome sequenced and the isolates were grouped into 19 different sequence types (STs) based on 7-gene MLST. A core genome (cg) MLST including 2360 genes clustered each ST separately (Fig. 1). There was an annual variation in the presence of STs; ST38 was the most common ST in both 2012 (*n* = 57, 86%) and 2014 (*n* = 16, 28%) but was not detected in 2016. A total of 241 allele differences were present among the 73 ST38 isolates, and none of the isolates displayed identical cgMLST profiles. ST1158 was also common among isolates collected in 2014 (*n* = 15, 26%). However, this ST was not present in either 2012 or 2016. Fifty allele differences were detected among isolates belonging to ST1158, and two isolates shared an identical cgMLST profile. In 2016, ST2040 emerged as a new ST, and was also the dominant ST that year (*n* = 11, 65%). A total of 32 allele differences were observed between the isolates belonging to ST2040, with two isolates displaying identical cgMLST profiles. The highest diversity of STs was observed in 2014, with 12 different STs being represented among the isolates, followed by seven different STs in 2012, and five in 2016. Two STs were present in all three years, namely ST10 (*n* = 3 + 2 + 1) and ST1594 (*n* = 1 + 1 + 1). In the cgMLST analysis, 39 and 44 allele differences were detected among ST10 and ST1594 isolates, respectively. None of the ST10 nor the ST1594 isolates shared identical cgMLST patterns (Fig. 1). An overview of the results from the ST-profiling is presented in Fig. 2.

### Virulence-associated geno- and phenotypes

In order to study the virulence potential of the 141 ESC isolates we used Virulence Finder and vfdb_core databases to detect virulence genes. Based on the authors’ knowledge and descriptions in the databases specific UPEC-associated virulence genes were identified. A complete overview of all virulence genes identified in each of the 141 isolates investigated is found in the Supplementary material (Table S1). In general, a limited number of genes encoding UPEC-associated virulence factors were present among the isolates. Genes encoding the UPEC-associated toxins hemolysin (*hly*) or cytotoxic necrotizing factor 1 (*cnf1*) were not identified in any of the isolates. The most common UPEC-associated virulence genes detected among the *E. coli* isolates encoded proteins involved in iron uptake, synthesis of type 1 fimbriae, serum survival, and capsule formation (Table S1). Furthermore, incomplete operons encoding certain traits, such as P-fimbriae (*papA-K*) and type 1 fimbriae (*fimA-I*) were identified in several isolates.

**Fig. 3:**
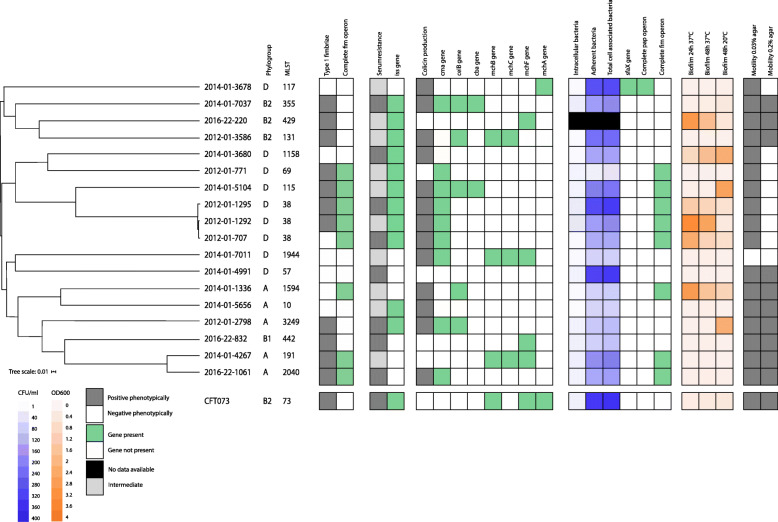
Phenotypic and genotypic virulence characteristics for 18 extended-spectrum cephalosporin-resistant *Escherichia coli* isolates originating from retail chicken meat in Norway. Overview of phenotypic virulence and presence/absence of selected virulence genes for the 18 isolates included in in vitro phenotypic experiments. Results of the phenotypic tests evaluating Type 1 fimbriae, serum resistance, colicin production, and motility are presented as positive (grey) or negative (white). Shades of blue represent cell adhesion and invasion. Shades of orange represent biofilm production. Presence and absence of selected virulence genes associated with the phenotypic assays are presented as green and white, respectively. Black boxes illustrate missing data

To evaluate the expression of detected genes and virulence-potential, a selection of isolates was chosen for further phenotypical testing. These isolates were selected based on the following criteria: 1) representatives from each phylogroup (A, B1, B2, or D) and different sequence types, 2) isolates with the most and the fewest virulence genes, 3) representatives from the most prevalent sequence types, 4) at least one representative from common ExPEC STs. An overview of these 18 isolates and their UPEC- and AMR-associated genes are summarized in Table 1. A summary of the results from the in vitro phenotypic tests related to the presence of selected virulence-associated genes is presented in Fig. 3. In total, there was limited consistency between the occurrence of genes and the corresponding traits detected in the in vitro phenotypic assays.

**Table 1:** Overview of genetic characteristics, including virulence and AMR genes, for 18 extended-spectrum cephalosporin-resistant *Escherichia coli* isolates included in the phenotypic characterization

ID	Year	ST	Phylo- group	Serotype	AMR genes	UPEC associated virulence genes
2012-01-1292	2012	38	D	O7:H18	*bla*_CMY-2_	*fimA-I*, *iucA-D*, *iutA*, *chuA*, *chuS-Y*, *entA-F*, *entS*, *fepA-D*, *fepG*, *iroN*, *kpsM*, *iss, cma,*
2012-01-1295	2012	38	D	O7:H18	*bla*_CMY-2_	*fimA-I*, *iucA-D*, *iutA*, *chuA*, *chuS-Y*, *entA-F*, *entS*, *fepA-D*, *fepG*, *iroN*, *kpsM*, *iss*, *iha, cma,*
2012-01-2798	2012	3249	A	O8:H9	*bla*_CMY-2_, *sul1*	*fimC-I*, *entA*, *entC*, *entE-F*, *entS*, *fepC-D*, *fepG*, *iss*, *astA, celB, cma*
2012-01-3586	2012	131	B2	O25:H4	*bla*_CMY-2_	*fimB-I, iucA-D*, *iutA*, *chuA*, *chuS-Y*, *entA-C*, *entE-F*, *entS*, *fepA-D*, *fepG*, *iroN*, *tsh*, *kpsM*, *iss*, *fyuA*, *iha*, *usp, celB, mchB, mchC, tsh*
2012–01-707	2012	38	D	O7:H18	*bla*_CMY-2_, *sul2*	*papB*, *papI*, *fimA-I*, *iucB-D*, *iutA*, *chuA*, *chuS-Y*, *entA-F*, *entS*, *fepA-D*, *fepG*, *iroN*, *kpsM*, *iss*, *iha, cma*
2012–01-771	2012	69	D	O17/O44, O17/O77:H18	*bla*_CMY-2_, *sul2*, *aadA*, *dfrA*	*fimB-D*, *fimF-I*, *iucA-D*, *iutA*, *chuS*, *chuU-Y*, *entA-C*, *entE-F*, *entS*, *fepA-D*, *fepG*, *iss*, *astA, cma*
2014-01-1336	2014	1594	A	O21:H4	*bla*_CMY-2_, *bla*_TEM1_	*fimA-I*, *iucA-D*, *iutA*, *entA-C*, *entE-F*, *entS*, *fepA-D*, *fepG*, *kpsM*, *astA, celB*
2014-01-3678	2014	117	D	O24:H4	*bla*_CMY-2_, *sul1*, *aadA*	*papB-papK*, *sfaX*, *fimB-I*, *iucB-D*, *iutA*, *chuA*, *chuS-Y*, *entA-C*, *entE-F*, *entS*, *fepA-D*, *fepG*, *iroN*, *pic*, *vat*, *ireA*, *fyuA, mcmA*
2014-01-3680	2014	1158	D	O17/O44, O17/O77:H34	*bla*_CMY-2_	*fimA-C*, *fimE-I*, *iucA-D*, *iutA*, *chuA*, *chuS-Y*, *entA-C*, *entE-F*, *entS*, *fepA-D*, *fepG*, *kpsM*, *iss*, *iha*
2014-01-4267	2014	191	A	O150:H20	*bla*_CMY-2_	*fimA-I*, *entA-C*, *entE-F*, *entS*, *fepA-D*, *fepG*, *iha, mchB, mchC, mchF*
2014-01-4991	2014	57	D	ONT:H18	*bla*_CMY-2_	*fimA-C*, *fimE-I*, *chuA*, *chuS-Y*, *entA-C*, *entE-F*, *entS*, *fepA-D*, *fepG*
2014-01-5104	2014	115	D	O102:H6	*bla*_CMY-2_	*fimA-I*, *iucB-D*, *iutA*, *chuV-Y*, *entA-F*, *entS*, *fepA-D*, *fepG*, *kpsM*, *iss*, *astA, cba, celB, cma*
2014-01-5656	2014	10	A	O125ab:H4	*bla*_CMY-2_	*fimB-I*, *iucB-D*, *iutA*, *entA-C*, *entE-F*, *entS*, *fepA-D*, *fepG*, *iss*, *fyuA*, *astA*, *iha*
2014-01-7011	2014	1944	D	O38:H39	*bla*_CMY-2_	*fimF-H*, *chuA*, *chuS-Y*, *entA-F*, *entS*, *fepA-D*, *fepG*, *iha, cma, mchB, mchC, mchF*
2014-01-7037	2014	355	B2	O2:O50/O2:H5	*bla*_CMY-2_	*fimB-I*, *iucA-D*, *iutA*, *chuA*, *chuS-Y*, *entA-C*, *entE-F*, *entS*, *fepA-D*, *fepG*, *kpsM*, *iss*, *fyuA*, *astA*, *iha*, *usp, cba, celB, cma*
2016–22-220	2016	429	B2	O50/O2:H1	*bla*_CMY-2_, *sul1*, *aadA*, *aac*, *tetA*	*fimA-I*, *iucB-D*, *iutA*, *chuA*, *chuS-Y*, *entA-F*, *entS*, *fepA-D*, *fepG*, *iroN*, *kpsM*, *iss*, *fyuA*, *usp, mchF*
2016–22-832	2016	442	B1	O91:H21	*bla*_CMY-2_, *dfrA*	*fimB-I*, *iucB-D*, *iutA*, *entA-C*, *entE-F*, *entS*, *fepA-D*, *fepG*, *iroN, mchF*
2016-22-1061	2016	2040	A	O159:H20	*bla*_CMY-2_	*fimA-I*, *iucB-D*, *iutA*, *entA-F*, *entS*, *fepA-D*, *fepG*, *iroN*, *tsh, cma*

Expression of the type 1 fimbriae was assessed by a yeast agglutination test which tests the ability of type 1 fimbriated bacteria to bind to mannose receptors on the surface of yeast cells. Seven out of 18 isolates did not agglutinate the yeast cells. Among the remaining 11 positive isolates, six harbored the complete *fim A- I* operon, while at least one of the genes in the *fim* operon was not detected in the other five. Among the seven isolates that were not able to agglutinate yeast cells, a complete *fim* operon was identified in two isolates (Fig. 3).

To be able to migrate from the gastrointestinal tract to other tissues, resistance to killing by human serum provides a huge advantage for bacterial survival. None of the isolates were classified as sensitive in the serum-resistance assay. Most of the isolates exhibited intermediate serum resistance (*n* = 10), whereas the remaining isolates were resistant to inactivation by human serum. Five of the resistant isolates and six of the isolates that exhibited intermediate resistance harbored the *iss* gene.

Production of colicins can provide competitive advantages by inhibiting growth of other coliform bacteria, ensuring the isolate to prevail. Thirteen isolates produced colicins. The most prevalent colicin-encoding gene detected among the *E. coli* isolates was *cma,* encoding Colicin M. The *cma* gene was present in eight of the colicin-producing isolates. In two of these isolates, *cma* was found in combination with *celB* (Colicin E2) and *cba* (Colicin B), in two isolates in combination with only *celB* or in combination with *mchB*, *mchC* and *mchF* (Microcin H47), respectively. One of the colicin-producing isolates carried only the *celB* gene, while *celB* was present in combination with *mchB* and *mchC* in another isolate. There was also one isolate that only carried the *mcmA* gene (Microcin M/Colicin V). Finally, there were two colicin-producing isolates which did not carry any of the investigated colicin-encoding genes. In four of the five isolates without colicin production, genes associated with colicin production were identified (Fig. 3).

We wanted to characterize the potential of our isolates to colonize a host. Thus, the ability to adhere to and invade Vero cells were studied. The isolates showed a high capability of bacterial adhesion to eukaryotic cells, but low capability of cell invasion. Data for cell adhesion and invasion for isolate 2016–22-220 (ST429) is not included in the results as concentrations of antibiotics used to kill adherent bacteria were not effective for this isolate, despite exceeding the predicted MIC values. Several genes may be involved in the process of cell adhesion. Genes investigated in this study include the *sfaX* gene*,* the *pap* operon and the *fim* operon. In only one of the isolates, 2014-01-3678 (ST117), both the *sfaX* gene and a complete *pap* operon were identified. However, an incomplete *fim* operon was also identified in the same isolate. This isolate displayed a high degree of cell adhesion (> 400 CFU/ml). The degree of cell adhesion among the eight isolates with a complete *fim* operon was observed to be very variable. Although isolate 2014-01-4991 (ST57) carried incomplete *fim-* and *pap* operons, and the *sfaX* gene was absent it showed a high degree of cell adhesion.

**Fig. 4:**
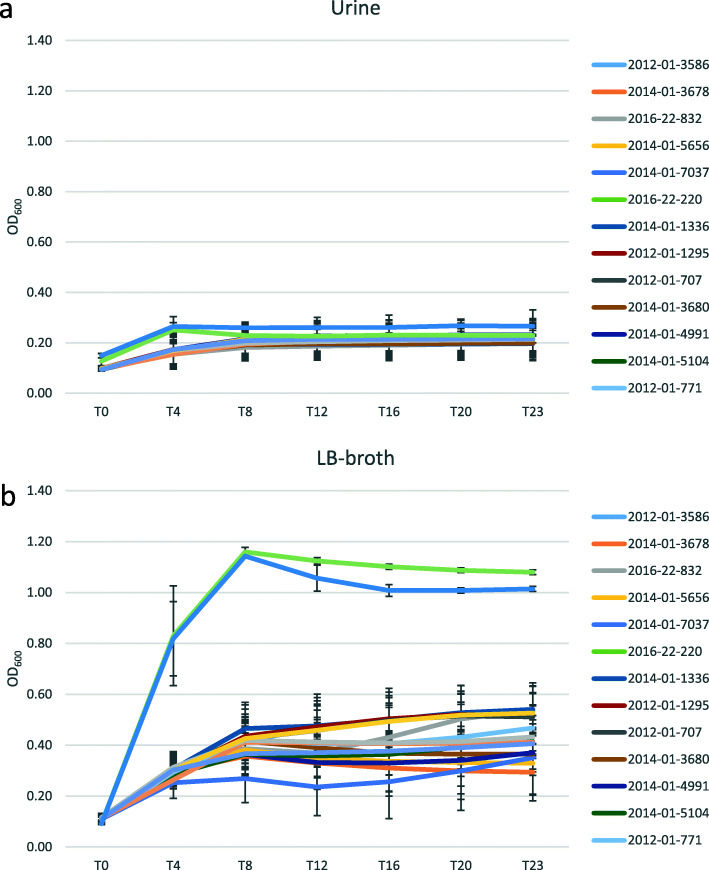
Bacterial growth. The figure illustrates the growth of the 18 *E. coli* isolates included in the in vitro phenotypic studies in sterile filtered human urine (**a**) and LB broth (**b**). Growth in LB was included as a measure on optimal growth conditions and used as basis for comparison with growth in sterile filtered human urine. Growth curves were obtained from a Tecan plate reader in which the optical density was measured at 600 nm every ten minutes for 23 h. Time is given in hours on the x-axis, and the measured optical density on the y-axis. The experiment was performed in triplicate, and the standard deviations are indicated by whiskers

Motility was evaluated as the ability of the bacteria to move through semi-liquid LB agar. None of the isolates were able to move in 0.7% agar. Eight of the isolates were non-motile in 0.2% agar, but only isolate 2014-01-7011 (ST1944) was non-motile in 0.03% agar. The ability to form biofilm varied among the isolates, between temperatures and culture incubation times. Biofilm production was defined by OD three times as high as the control. Isolate 2012–01-707 (ST38) produced the strongest biofilm at 37 °C, while isolate 2016-22-1061 (ST2040) was the strongest biofilm producer at 20 °C. Nine of the isolates displayed poor biofilm formation at both 37 °C and 20 °C. All isolates were able to grow in human urine (Fig. 4). Isolate 2016-22-1061 (ST2040) exhibited the most rapid growth of the 18 isolates, given the experimental conditions provided, both in urine and in LB. Growth rates are provided in the supplementary material (Table S2).

### Acquired AMR genes

The presence of *bla*_CMY-2_ in all isolates was confirmed by the WGS data, and some of the isolates carried additional resistance genes. These included ampicillin-resistance gene *bla*_TEM1B_ (*n* = 13), sulfonamide-resistance genes *sul1* (*n* = 5) and *sul2* (*n* = 11), streptomycin- and spectinomycin-resistance gene *aadA1* (*n* = 6), aminoglycoside-resistance genes *aac (3)-*VIa (*n* = 3) and *aph* (*n* = 2), tetracycline-resistance genes *tet*(A) (*n* = 3) and *tet*(B) (*n* = 2), and trimethoprim-resistance genes *dfrA1* (*n* = 2) and *dfrA5* (*n* = 1). In general, isolates with the same ST had highly similar AMR genes. An overview of acquired AMR genes and their association with ST in all included isolates is presented in the Supplementary material (Fig. S1 and Table S1).

## Discussion

We have characterized by WGS all ESC-resistant *E. coli* isolated from retail chicken in Norway in 2012, 2014, and 2016 obtained through the NORM-VET monitoring program. This enabled in-depth characterization of the population dynamics among ESC-resistant isolates over a prolonged time period. As mentioned earlier NORM-VET is governed by the legislation for surveillance of AMR, and the sampling was therefore performed following defined schemes designed to be representative for the entire population [21].

Since 2012 there has been a significant decrease in the occurrence of ESC-resistant *E. coli* in Norwegian retail chicken [40]. Newly published data showed that only 0.4% of samples were positive in 2018 [40]. Furthermore, semi-quantitative methods have revealed that in the vast majority of Norwegian retail chicken samples where ESC-resistant *E. coli* were detected, only very low levels of these bacteria were present (≤ 0.2 cfu/g) [19, 41]. This is consistent with recent trends reported from Denmark, Sweden, and the Netherlands, where a decrease in the number of ESC-resistant isolates has also been described over the last decade [42–44]. In 2014, the Norwegian poultry industry initiated an action plan against antimicrobial resistant bacteria in broiler production [22]. The sum of measures taken by the industry, both nationally and internationally, has likely contributed to improve the situation.

Although the occurrence of ESC-resistant *E. coli* in Norwegian retail chicken decreased during the five-year study period, the relatively large variation in STs indicates that there are annual fluctuations in the population of ESC-resistant *E. coli* STs. This underlines the complex epidemiology of ESC-resistant *E. coli*, which has also been highlighted by others [45]. Understanding the epidemiology is further complicated by the occurrence of both vertical and horizontal dissemination of ESC-resistance. The vast majority of ESC-resistant *E. coli* included in this study were known to carry *bla*_CMY-2_ on self-transferable IncK plasmids [46]. However, in the emerging ST2040, *bla*_CMY-2_ was present on a non-transferable IncI1 plasmid (unpublished data). We have not investigated if virulence genes were located on plasmids or a possible co-location of AMR- and virulence genes. In order to do so, it is necessary to perform long-read sequencing to enable reliable hybrid assemblies. This would enable us to consider the potential of dissemination and co-dissemination of AMR- and virulence determinants in the bacterial population.

Only two STs were present over all three years, namely ST10 and ST1594. However, only one or a limited number of isolates belonging to these STs were detected each year, indicating that they were not common in the ESC-resistant *E. coli* population. The three major STs in our material, ST38, ST1158, and ST2040, have all been previously described to occur in the broiler production in Europe [47–49]. This observation intrigues us to claim that certain successful ESC-resistant *E. coli* STs disseminate widely in the European broiler production, but further comparisons of sequence- and epidemiological data are warranted to confirm this hypothesis.

Five of the 19 *E. coli* STs identified among our isolates (ST131, ST117, ST38, ST10, and ST69) are included in the “top 20 ExPEC ST”-list published in a meta-analysis [50]. This included 217 studies that performed MLST or whole-genome sequencing to genotype *E. coli* recovered from extraintestinal infections or the gut. Our results from analyses of virulence associated genes and in vitro virulence assays revealed a large variation in the estimated virulence potential among the different STs including those that previously have been classified as ExPEC. The high diversity of virulence-associated traits suggests that the uropathogenic potential of ESC-resistant *E. coli* from poultry meat is isolate dependent and/or dependent on the sensitivity of the individual host. Based on data from the in vitro virulence assays, none of the isolates belonging to known ExPEC STs appeared to have higher uropathogenic potential than isolates belonging to the other STs. However, host related factors have not been considered in this work, and thus may affect the clinical outcome in an infection.

An important prerequisite for developing a UTI is the ability of uropathogenic *E. coli* to adhere to and invade the uroepithelium, and thus colonize the mucosal epithelial surfaces in the urinary tract. Expression of type 1 fimbriae which mediate bacterial adhesion to mannose-containing structures on the uroepithelium is particularly associated with UTIs [51], and this feature was found in half of our selected isolates. Notably, several of the isolates were able to form functional type 1 fimbriae despite lacking a complete *fim-*operon. None of the isolates showed a high degree of invasiveness in Vero cells under the experimental conditions used, and this could indicate that long-term survival in the uroepithelium would be limited.

We evaluated biofilm formation, colicin production and ability to grow in urine, all of which may influence the ability of isolates to colonize the human urinary tract [52, 53]. All isolates were able to grow in human urine, which may be a predictor of the colonization ability of uropathogens [54]. This is consistent with growth of *E. coli* CFT073 performed in urine by others [55, 56].

One of the first steps of colonization is the establishment of biofilm, which provides protection from the shear forces of passing urine in the urinary tract. In addition, the forming of biofilm will also provide advantages in avoiding the immune system and giving increased protection from antimicrobials [57]. The ability of the isolates to form biofilm in Luria-Bertani (LB) broth without NaCl was tested at two different temperatures, and at two different time points. Biofilm formation is highly dependent on environmental conditions and the access to nutrients. Half of the tested isolates were able to form biofilm and most isolates of those that were positive formed more biofilm at 37 °C compared to 20 °C, possibly indicating ability to form biofilm in the human body. Previous reports have indicated that uropathogenic bacteria within biofilms or in biofilm-like communities may promote virulence under certain growth conditions, for example by creating intracellular pod-like bulges inside of the bladder epithelial cells [58, 59]. This is considered important especially for recurrent UTIs, where the intracellular bacterial communities (IBC) facilitate persistence in the urinary epithelium [58, 59]. Our study provided limited experimental conditions, where the ability to form IBCs was not studied. Further studies of the ability of ESC-resistant *E. coli* to form biofilm on human urinary tract epithelium are needed to evaluate their uropathogenic potential. The production of specific enzymes and toxins, for example colicins, can provide beneficial colonization conditions as it limits competition from other bacteria [52]. As most of our isolates produced colicins, we can assume that these isolates possess competitive advantages over other *E. coli* isolates in the intestines.

Serum resistance is a key virulence trait of isolates that cause urosepsis and all isolates tested were serum resistant. Urosepsis is a serious complication in UTIs that requires immediate medical care to avoid a possible life-threatening situation [60]. Furthermore, if an UTI is caused by an UPEC isolate resistant to clinically relevant antimicrobials, the treatment could be complicated, prolonged and costly [61, 62].

Isolate 2016-22-1061 (ST2040) stood out as the isolate which expressed the most UPEC-associated virulence factors (type 1-fimbriae, production of colicins, survival in human serum, and the fastest growth in urine). Interestingly, this isolate belongs to phylogroup A, which is rarely described to cause extraintestinal infections. Nevertheless, due to its estimated pathogenicity and recent emergence as described above, attention should be paid to this ST in future surveillance.

Isolate 2012-01-3586 belonged to ST131, serotype O25:H4 and phylogroup B2, which is known to be a notorious ExPEC ST and is considered a high-risk clone [63, 64]. Surprisingly, this isolate appeared have a lower virulence potential than expected; it produced colicin, expressed the type 1-fimbriae and adhered to eukaryotic cells, but it was among the isolates that expressed the lowest serum resistance. It did not invade Vero cells, and was a weaker biofilm producer compared to all isolates tested. Furthermore, none of the genes encoding toxins commonly produced by pathogenic ST131 isolates, namely *pic*, *vat*, *sat*, *hlyA/D*, *astA*, *cdtB,* and *cnf1* [65, 66] were present in the genome of this isolate. We detected the *fimH*38 allele in isolate 2012-01-3586, while the global high-risk ST131 clone has been associated with the *fimH*30 allele [63, 67]. Thus, it is possible that this isolate belongs to a sub-group with lower pathogenic potential compared with the previously described ST131 ExPEC clone.

The lack of direct correlation between the observed genotypes and phenotypes in our experiments complicates interpretation of the results. Many of the traits that we investigated have complex genetic backgrounds, and several genes may give rise to the same phenotype. For example; isolate 2014-01-4991 (ST57) carried incomplete *fim-* and *pap* operons, and the *sfaX* gene was absent but still showed a high degree of cell adhesion in the phenotypic testing. This genotype to phenotype divergence illustrates the importance of performing in vitro virulence tests in order to assess the possible pathogenic potential of isolates rather than relying solely on comparative genomics [68, 69]. Furthermore, the study of virulence associated traits was limited to only 18 isolates, but discrepancy observed between genotype and phenotype indicates that there could be differences in pathogenic potential within the same ST. For comparison of phenotypic characteristics of relevance for pathogenicity, we used *E. coli* CFT073 as a positive control strain in all our in vitro experiments. This strain was originally isolated from the blood of a woman with acute pyelonephritis and is regarded as an UPEC prototype [70]. However, none of the isolates that we investigated had an identical phenotypic profile to that of *E. coli* CFT073 (Fig. 3).

The plasmid mediated AmpC beta-lactamase encoding gene *bla*_CMY-2_ has played a major role in conferring ESC-resistance in the Norwegian *E. coli* isolates, while genetic linkages to genes encoding resistance to other antimicrobial classes have not been prominent [46, 71]. Previous studies of the phenotypical resistance patterns of these isolates have confirmed that the occurrence of co-resistance to other antimicrobials is limited [41, 71, 72]. In this study we focused on ESC-resistant *E. coli.* However, AMR and virulence genes are not necessarily linked, and it is possible that susceptible *E. coli* of broiler origin may have a different virulence potential than the ESC-resistant isolates investigated here. Several studies have suggested that food products, especially chicken meat, are an important source of ESC-resistant ExPEC [31, 36]. On the other hand, other more recent studies have reported a limited contribution of chicken meat to the overall occurrence of ESC-resistant *E. coli* in humans [73–76]. By revisiting previously analyzed materials using WGS, de Been et al. failed to provide any evidence for recent clonal transmission of ESC-resistant *E. coli* strains from poultry to humans [77]. All these studies were performed in countries where the occurrence of ESC-resistant *E. coli* in poultry and/or chicken meat is higher than in Norway. The occurrence of ESC-resistance among clinical UPEC isolates in Norway is considered very low, only 3.4% in 2018 [40]. However, results from one Norwegian study indicated that clonal transfer of ESC-resistant *E. coli* from chicken meat to humans may occur, and that these bacteria could be a source of ESC-resistance plasmids that could be transferred to bacteria residing in the human gut microbiota [78]. However, only a limited number of isolates were included in the Norwegian study, and the presence of virulence factors known to be associated with UTIs was not in focus.

One strength of our study is that all ESC-resistant *E. coli* isolated from retail chicken in Norway in 2012, 2014, and 2016 were characterized in depth using cgMLST and that acquired AMR genes and virulence genes were investigated. As well as providing an overview of the population structure, these data also demonstrate the occurrence or absence of genes that encode virulence factors of possible relevance regarding pathogenic potential.

## Conclusion

Our study showed a fluctuation in the ST composition of ESC-resistant *E. coli* isolated from retail chicken meat in 2012, 2014, and 2016. Five of the STs present have previously been associated with ExPEC. However, results from the in vitro virulence assays did not indicate that our isolates from these STs had a higher pathogenic potential than isolates from other STs. These observations suggest that the estimated pathogenic potential of ESC-resistant *E. coli* from poultry meat is highly dependent on the individual isolate. In conclusion, our results indicate that the uropathogenic potential of ESC-resistant *E. coli* from the Norwegian poultry reservoir is limited. It is reasonable to assume that the risk of being exposed to ESC-resistant *E. coli* with pathogenic potential through handling and consumption of chicken meat in Norway is low. However, we have also shown that the population structure of the ESC-resistant *E. coli* is dynamic and the genetic diversity of the fluctuating STs is considerable. It is therefore important to maintain monitoring programs and the implementation of preventive measures to hinder the emergence of AMR and potential pathogenic variants in the Norwegian broiler production.

## Materials and methods

### Bacterial isolates

All ESC-resistant *E. coli* isolated from domestically produced retail chicken meat in the NORM-VET program in 2012 (*n* = 66), 2014 (*n* = 58), and 2016 (*n* = 17) were included in the study (total *n* = 141). All isolates were known to carry the *bla*_CMY-2_ gene encoding ESC resistance [19, 41, 72]. Year of isolation, phylogroup, serotype, and AMR profile of the 18 isolates selected for in vitro virulence characterization are described in Table 1. *E. coli* CFT073 [79], a known UPEC strain, was also analyzed for comparison in the in vitro experiments.

#### DNA extraction

Total genomic DNA was extracted using the DSP DNA Mini Kit (Qiagen, Hilden, Germany) and the QIAsymphony automated extractor (Qiagen), or manually either using the QIAmp DNA Mini kit or the Qiagen Blood and Tissue kit (Qiagen). DNA concentration was determined on a Qubit™ fluorometer (ThermoFischer Scientific, Waltham,) using the Qubit™ dsDNA BroadRange assay kit (ThermoFischer Scientific). DNA purity was measured on a Nanodrop 2000 spectrophotometer (ThermoFischer Scientific).

#### Whole genome sequencing

Samples were prepared with either the Nextera XT or Nextera Flex library preparation kit (Illumina, San Diego, CA, USA). Whole genome sequencing was performed on an Illumina HiSeq X (*n* = 12) or Illumina NextSeq 500 (*n* = 107), resulting in 150 bp paired-end reads, or on a HiSeq 2500 using rapid mode (*n* = 9), resulting in 250 bp paired-end reads. In addition, sequence data for 13 isolates (five from 2012 and eight from 2014) had been sequenced previously [46, 78], and raw reads were available for inclusion in the present study. An overview of which isolates were sequenced on the different platforms is provided in the Supplementary material (Table S1).

### Bioinformatics

Initial quality control and assembly of samples was done using the Bifrost pipeline [80]. Briefly, quality control of the paired end reads was done using the FastQC tool (https://www.bioinformatics.babraham.ac.uk/projects/fastqc/) and the results were merged using MultiQC [81]. Further, PhiX was removed by BBDuk (https://sourceforge.net/projects/bbmap/), and sequences were trimmed with Trimmomatic [82]. Assembly was done using SPAdes [83], polished using Pilon [84], and the quality of the assemblies evaluated with QUAST [85].

Subsequently, the Bifrost pipeline for identification of specific genes such as MLST, AMR genes and virulence genes were used [86] In detail, the ARIBA (Antimicrobial Resistance Identification By Assembly) software [87] was used to determine multilocus sequence type (MLST) according to the Achtman scheme [88]. The presence of acquired resistance genes as well as virulence genes was determined using the ResFinder [89] and VirulenceFinder [90] and vfdb_core [91] databases, respectively. The analyses were performed in September 2019 and the updated databases were used. The *E. coli* serotypes were determined using SerotypeFinder [92].

Results from ARIBA were summarized using VAMPIR- Virulence, AMR, MLST and Plasmid analysis in R (https://github.com/hkaspersen/VAMPIR, commitid 54d687a) in R version 3.5.2 [93].

All isolates were subjected to cgMLST analysis, including 2360 genes, in order to investigate the genetic relationship between isolates. This was done using the cgMLST scheme available from Enterobase (https://enterobase.warwick.ac.uk/) in the chewBBACA suite [94]. The cgMLST tree was visualized using the ggtree package [95] in R version 3.5.2 [93]. Sequence data is available at European Nucleotide Archive (ENA). Accesion numbers are given in Table 2.

**Table 2:** Accession numbers for sequence data at European Nucleotide Archive (ENA)

Group	Read length	Setsize	N	Project accession
Nextseq	150	2	107	PRJEB40941
HiseqX	150	2	12	PRJEB40952
Hiseq2500	100	2	13	PRJEB40969
Hiseq2500rapid	250	4	9	PRJEB41003

#### In vitro virulence expression

Results from the genetic analysis and in vitro testing were summarized and visualized using iTOL (http://itol.embl.de) [96], and are presented in Fig. 3.

##### Expression of type 1 fimbriae

The ability to express a D-mannose-binding phenotype, characteristic for functional Type 1 fimbriae, was assayed by the ability to agglutinate yeast cells (*Saccharomyces cerevisiae)* [97]. Each isolate was inoculated into LB broth and incubated over night at 37 °C. One ml of overnight culture was centrifuged (3000 x g, 5 min) and the pellet resuspended in 100 μl PBS. Ten μl of the bacterial suspension was mixed with 10 μl yeast cells (5 mg/ml, resuspended in PBS) with and without 1% D-mannose solution on a microscopy slide, and agglutination observed visually. Suspension containing 1% D-mannose was considered a negative control.

##### Motility test

One colony from a fresh blood agar plate of each isolate was perpendicularly inoculated into a tube containing 5 ml semi-solid LB agar, at concentrations of 0.03, 0.2, and 0.7% agar, and incubated for 24 h at 37 °C [98]. Motile bacteria appeared as a “cloud” of bacterial growth in the agar around the stab-line.

##### Biofilm production

Biofilm production was evaluated as described by Stromberg et al., with minor modifications [99]. Briefly, overnight cultures in LB broth were diluted 1:200 in LB without NaCl, and 200 μl added to a 96-well microtiter plate (Greiner, Sigma-Aldrich, Germany). Wells containing uninoculated media were used as negative controls. The plates were incubated at 37 °C for 24 h and at 20 °C for 48 h. After incubation, the plates were washed three times with PBS to remove planktonic cells. Adhered bacteria were stained with 0.1% crystal violet for 15 min, followed by washing three times with PBS. Thereafter, 200 μl ethanol was added to each well and OD_600_ was measured using Infinite M200 plate reader (Tecan, Männedorf, Switzerland). Biofilm formation was considered when the OD_600_ was at least three times greater than that of the negative control [99].

##### Bacterial growth

Overnight cultures of each isolate were diluted 1:1000 in fresh LB broth. Thereafter, 200 μl of each isolate was transferred to a 96-well microtiter plate (Greiner, Sigma-Aldrich, Germany) and incubated at 37 °C in Infinite M200 plate reader (Tecan). OD_600_ was measured every 10 min for 24 h. The experiment was repeated three times for each isolate.

In addition, bacterial growth was tested in sterile-filtered human urine (pH = 6.5) with the same protocol as for LB. Urine was collected from healthy female volunteers with no history of UTI or antibiotic use in the previous two months.

##### Serum resistance

In order to investigate resistance to human serum, 250 μl of the overnight cultures were added to 750 μl 20% human serum (Sigma-Merck) (HS, diluted in PBS) or heat-inactivated serum (HIS, control for comparison). Serum was inactivated by incubating in a water bath at 56 °C for 60 min. The mixtures were incubated at room-temperature, and samples were taken every hour for three hours. Samples were serially diluted and plated on LB agar plates. The plates were incubated for 24 h at 37 °C and colonies counted. The colonies from HS samples were calculated as a percentage of the HIS samples. The results were categorized as follows: < 1% = serum sensitive, > 90% = serum resistant, and all other results were considered as intermediate [100].

##### Adhesion to and invasion of eukaryotic cells

Adhesion to, and invasion of, cells was tested in Vero cells (Vero C1008, ECACC, Item number 85020206) grown at 37 °C [101]. The cells were grown to 80% confluence, and 200 μl of cells in fresh minimal essential medium (DMEM (GibcoTM 11,568,876)) with 10% Fetal Bovine Serum (GibcoTM 10,270,106) and 1 ml penicillin/streptomycin solution (GibcoTM, 15,140,122, containing 10,000 units/ml penicillin and 10,000 μg/ml streptomycin) added to 100 ml DMEM was transferred to a microtiter plate (Greiner, Sigma-Aldrich, Germany). This was done in duplicate, at a concentration of approximately 5*10^4^ cells/ml (counted in Countess (Thermo Scientific,), and the cells grown to confluence. Overnight cultures of bacteria were diluted 1:100 in fresh LB broth to OD_600_ = 0.1. One ml was centrifuged at 500 x g for 5 min and the pellet was resuspended in 500 μl fresh DMEM cell-medium without antibiotics. The bacterial suspension was diluted 1:100 in DMEM cell-medium without antibiotics and 50 μl was added to the confluent Vero cells with fresh cell medium, equivalent to approximately 30 bacteria per cell (MOI 30:1). The plates were centrifuged at 100 x g for 2 min to increase contact between bacteria and cells, and incubated for two hours at 37 °C.

To assess adhesion to cells, the cells were washed three times with PBS to remove non-adherent bacteria and lysed with 30 μl 1% Triton X for 10 min. The lysates were serially diluted in PBS and plated on LB agar. To assess bacterial invasion, 200 μl of fresh medium with antibiotics (0.1 mg/ml gentamicin and 20 mg/ml nalidixic acid) was added to the cells before incubation at 37 °C for two hours to kill adherent bacteria. The cells were lysed and plated as described for the adhesion assay.

##### Colicin production

Colicin production was investigated as described previously [102]. Briefly, 100 μl overnight culture of *E. coli* DH5α was spread onto LB agar plates and left to dry for approximately 10 min at room temperature. One ml overnight culture of the respective isolates was centrifuged at 13000 x g for 10 min and the supernatants sterile filtered through a 0.22-μm Minisart® syringe filter (Sartorius Stedim Biotech GmbH, Germany). Ten μl of the filtrate was spot inoculated on the dried LB agar plates with *E. coli* DH5α and incubated at 37 °C for 24 h. Production of colicin was determined by the presence of an inhibition zone around the place of inoculation.

##### Statistical analysis

All assays were performed in three parallels for each isolate, and each experiment was repeated three times. Standard deviations (SDs) for quantitative data were calculated (supplementary material).

## Supplementary Information

**Additional file 1.**

**Additional file 2.**

## Data Availability

The datasets used and/or analyzed during the current study are available from the corresponding author on reasonable request. Sequence data is available at European Nucleotide Archive (ENA). Accession numbers are given in Table 2.
