# Supplementary material for: Population structure and uropathogenic potential of extended-spectrum cephalosporin-resistant Escherichia coli from retail chicken meat
Source: BMC Microbiol. 2021 Mar 29;21:94. doi: 10.1186/s12866-021-02160-y (PMC8008618; doi:10.1186/s12866-021-02160-y)
Supplement: Supplementary file 1 — Additional file 1. [file 12866_2021_2160_MOESM1_ESM.docx]

*Supplementary material*

**Population structure and uropathogenic potential of extended-spectrum cephalosporin-resistant *Escherichia coli* from retail chicken meat**

**May Linn Buberg^a#^, Solveig Sølverød Mo^b#^, Camilla Sekse ^b^, Marianne Sunde ^b^, Yngvild Wasteson^a^, Ingun Lund Witsø ^a*^**

^a^Department of Paraclinical Sciences, Faculty of Veterinary Medicine, Norwegian University of Life Sciences, Oslo, Norway

^b^Section for Food safety and Animal Health Research, Department of Animal Health and Food Safety, Norwegian Veterinary Institute, Oslo, Norway

# Both authors contributed equally to this work

**Running title:** Uropathogenic potential of *E. coli* from poultry

***Corresponding author:**

Ingun Lund Witsø, ingun.lund.witso@nmbu.no, Phone: +47 958 20 913

**Supplementary**

**
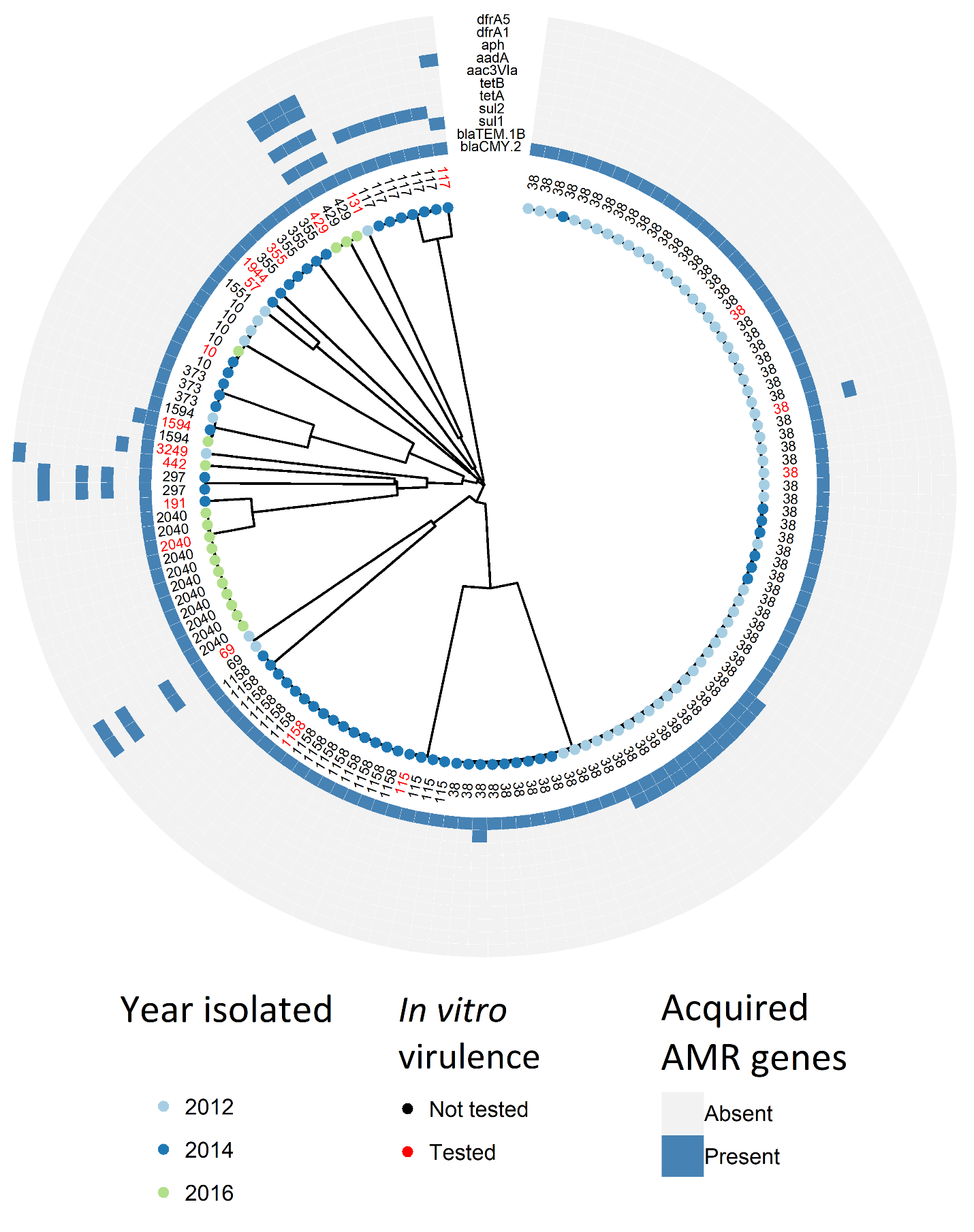
**

**Figure S1 – Clustering of 141 extended-spectrum cephalosporin-resistant *Escherichia coli* isolated from Norwegian retail chicken meat.**

The clustering is based on core genome multilocus sequence typing. Year isolated is indicated as light blue (2012), blue (2014), or light green (2016) dots. The 18 isolates marked in red were included in the *in vitro* virulence assays. Presence of acquired AMR genes is illustrated with blue boxes, while absence is illustrated with grey boxes.

**Table S1 – Total dataset**All isolates and their respective genetic profile, in addition to sequence method performed for each of the respective isolates.

**Table S2 – Growth curves supplementary**Growth rate, doubling time, and R^2^ for each isolate for growth in LB-broth and sterile-filtered human urine. The optical density was measured at 600 nm in a Tecan plate reader every ten minutes for 23 hours. This was done in triplicate, and the average growth rate and doubling time were calculated in Excel.

| **Medium** | **Isolate** | **Growth rate** | **Doubling time** | **R^2^** |
| --- | --- | --- | --- | --- |
| LB | 2012-01-3586 | 0.216 | 3.20 | 0.667 |
|  | 2014-01-3678 | 0.101 | 6.81 | 0.296 |
|  | 2016-22-832 | 0.206 | 3.34 | 0.727 |
|  | 2014-01-5656 | 0.120 | 5.73 | 0.359 |
|  | 2014-01-7037 | 0.135 | 5.11 | 0.616 |
|  | 2016-22-220 | 0.212 | 3.26 | 0.657 |
|  | 2014-01-1336 | 0.208 | 3.31 | 0.638 |
|  | 2012-01-1295 | 0.212 | 3.25 | 0.064 |
|  | 2012-01-707 | 0.210 | 3.28 | 0.659 |
|  | 2014-01-3680 | 0.135 | 5.11 | 0.417 |
|  | 2014-01-4991 | 0.130 | 5.30 | 0.441 |
|  | 2014-01-5104 | 0.164 | 4.21 | 0.590 |
|  | 2012-01-771 | 0.181 | 3.82 | 0.59 |
|  | 2014-01-7011 | 0.169 | 4.08 | 0.560 |
|  | 2014-01-4267 | 0.160 | 4.31 | 0.521 |
|  | 2012-01-1292 | 0.210 | 3.29 | 0.663 |
|  | 2012-01-2798 | 0.157 | 4.38 | 0.546 |
|  | 2016-22-1061 | 0.281 | 2.46 | 0.427 |
|  | E. coli CFT073 | 0.268 | 2.58 | 0.404 |
| Urine | 2012-01-3586 | 0.099 | 7.00 | 0.570 |
|  | 2014-01-3678 | 0.112 | 6.14 | 0.648 |
|  | 2016-22-832 | 0.916 | 0.75 | 0.644 |
|  | 2014-01-5656 | 0.101 | 6.85 | 0.609 |
|  | 2014-01-7037 | 0.107 | 6.47 | 0.649 |
|  | 2016-22-220 | 0.104 | 6.64 | 0.613 |
|  | 2014-01-1336 | 0.091 | 7.55 | 0.571 |
|  | 2012-01-1295 | 0.106 | 6.52 | 0.591 |
|  | 2012-01-0707 | 0.116 | 5.94 | 0.594 |
|  | 2014-01-3680 | 0.092 | 7.52 | 0.587 |
|  | 2014-01-4991 | 0.119 | 5.81 | 0.607 |
|  | 2014-01-5104 | 0.112 | 6.13 | 0.618 |
|  | 2012-01-771 | 0.110 | 6.25 | 0.600 |
|  | 2014-01-7011 | 0.112 | 6.14 | 0.679 |
|  | 2014-01-4267 | 0.101 | 6.81 | 0.599 |
|  | 2012-01-1292 | 0.110 | 6.29 | 0.589 |
|  | 2012-01-2798 | 0.105 | 6.55 | 0.553 |
|  | 2016-22-1061 | 0.057 | 12.15 | 0.287 |
|  | E. coli CFT073 | 0.062 | 11.03 | 0.398 |
